# Are radiomics features universally applicable to different organs?

**DOI:** 10.1186/s40644-021-00400-y

**Published:** 2021-04-07

**Authors:** Seung-Hak Lee, Hwan-ho Cho, Junmo Kwon, Ho Yun Lee, Hyunjin Park

**Affiliations:** 1 Departement of Electronic Electrical and Computer Engineering, Sungkyunkwan University, Suwon, 16419 South Korea; 2 Center for Neuroscience Imaging Research, Institute for Basic Science (IBS), Suwon, 16419 South Korea; 3 Core Research & Development Center, Korea University Ansan Hospital, Ansan, 15355 South Korea; 4 Department of Radiology and Center for Imaging Science, Samsung Medical Center, Sungkyunkwan University School of Medicine, 81 Irwon-Ro, Gangnam-Gu, Seoul, 06351 South Korea; 5 Department of Health Sciences and Technology, SAIHST, Sungkyunkwan University, Seoul, 06351 South Korea; 6 School of Electronic and Electrical Engineering, Center for Neuroscience Imaging Research, Sungkyunkwan University, Suwon, 16419 South Korea

**Keywords:** Radiomics, Macroscale tumor features, Tumor microenvironment, Computed tomography, Magnetic resonance imaging, Survival analysis

## Abstract

**Background:**

Many studies have successfully identified radiomics features reflecting macroscale tumor features and tumor microenvironment for various organs. There is an increased interest in applying these radiomics features found in a given organ to other organs. Here, we explored whether common radiomics features could be identified over target organs in vastly different environments.

**Methods:**

Four datasets of three organs were analyzed. One radiomics model was constructed from the training set (lungs, *n* = 401), and was further evaluated in three independent test sets spanning three organs (lungs, *n* = 59; kidneys, *n* = 48; and brains, *n* = 43). Intensity histograms derived from the whole organ were compared to establish organ-level differences. We constructed a radiomics score based on selected features using training lung data over the tumor region. A total of 143 features were computed for each tumor. We adopted a feature selection approach that favored stable features, which can also capture survival. The radiomics score was applied to three independent test data from lung, kidney, and brain tumors, and whether the score could be used to separate high- and low-risk groups, was evaluated.

**Results:**

Each organ showed a distinct pattern in the histogram and the derived parameters (mean and median) at the organ-level. The radiomics score trained from the lung data of the tumor region included seven features, and the score was only effective in stratifying survival for other lung data, not in other organs such as the kidney and brain. Eliminating the lung-specific feature (2.5 percentile) from the radiomics score led to similar results. There were no common features between training and test sets, but a common category of features (texture category) was identified.

**Conclusion:**

Although the possibility of a generally applicable model cannot be excluded, we suggest that radiomics score models for survival were mostly specific for a given organ; applying them to other organs would require careful consideration of organ-specific properties.

**Supplementary Information:**

The online version contains supplementary material available at 10.1186/s40644-021-00400-y.

## Background

Modern oncology is moving toward pursuing precision medicine, and medical imaging is an important factor in such an environment [1]. Traditionally, medical imaging has been interpreted by trained expert radiologists using imaging features. A few imaging features (e.g., tumor volume and shape) were used to assess tumor properties of various target organs [2–4]. Recently, medical imaging has been analyzed using a radiomics approach, which considers hundreds or even thousands of mineable features [5, 6]. With so many available features, radiomics has the potential to provide personalized information for various organs over different diseases [5, 7, 8]. There have been many successful radiomics studies reporting better performance than conventional imaging studies [9–11].

Macroscale features of the tumor such as size, shape, and texture are reflected in radiomics features [5, 12]. These features were useful in quantitively describing various aspects of tumor especially for evaluating regional heterogeneity. The tumor margin is an extremely dynamic area composed of immune cells, rich vasculature, lymphatics, fibroblasts, pericytes, and adipocytes, related to the tumor microenvironment [13–15]. It plays a vital role in prognosis and therapy response, especially for immunotherapy [16, 17]. Hence, the radiomics features in the marginal region might reflect the tumor microenvironment. Radiomics studies have identified essential features that non-invasively capture information related to the tumor microenvironment. In particular, quantitative imaging features based on histogram, texture, and shape provide information about the tumor microenvironment [5, 18]. One study showed that homogeneity, dissimilarity, contrast, and energy from the texture category of features reflected the local immune microenvironment of non-small cell lung cancer (NSCLC) [15]. Another study identified homogeneity and difference entropy from the texture category as biomarkers that could predict immune response [19]. The identified radiomics features are largely specific to the organ being modeled, the associated macroscale features and tumor microenvironment, imaging modality, and acquisition parameters. Still, many studies evaluated models trained in one setting to another setting possibly in different organs [17, 18]. This is because tumors in different organs might share common properties. Thus, there is a scientific curiosity in applying radiomics features found in a given organ to other tumors in different organs [20, 21]. A seminal study by Aerts et al. explored the application of the same radiomics features to two different organs, but this topic was not their primary aim and was thus insufficiently explored. A seminal study explored the application of the same radiomics features to two different organs (i.e., lung and head & neck cancers) and was successful at classifying the risk groups [20]. Furthermore, the study had issues regarding how the features were selected. A recent study explored the feasibility of applying the same set of radiomics features to intrahepatic cholangiocarcinoma, osteosarcoma, and pancreatic neuroendocrine tumors [21]. This translates to finding a “universal” set of radiomics features that can be applied to potentially different tumor microenvironments, in which they succeeded. Therefore, there is a need to investigate this issue further.

To this end, our study aimed to explore whether common radiomics features can be identified across different target organs. We evaluated the radiomics features of tumors in three organs (i.e., lungs, kidneys, and brains). We trained a radiomics model and chose features using lung data for survival analysis. The chosen features were applied to three independent test datasets of lung, kidney, and brain tumors, and the possibility of using the features to separate high- and low-risk groups was explored.

## Methods

### Patients and imaging datasets

Retrospective analysis of publicly available datasets was performed after receiving approval from the Institutional Review Board at the Sungkyunkwan University, Korea. This study considered four independent datasets: two lung cancer, one kidney cancer, and one brain cancer datasets. We considered only primary cancers of each dataset in this study. Table 1 and supplementary material provide a detailed description of each dataset.

**Table 1:** Summary of all datasets

	Training set	Test set1	Test set2	Test set3
**Reference**	NSCLC radiomics	TCIA lung CT diagnosis	TCGA-KIRC	CPTAC-GBM
**Organ**	Lung	Lung	Kidney	Brain
**Modality**	CT	CT	CT	MRI
**Number of patients**	422 (M: 290, F:132)	61 (M: 31, F:30)	48 (M:29, F: 19)	43 (M: 31, F:12)
**Age**	68.1 years (Avg.)	≥ 65 years: 41; < 65 years: 20	65.3 years	63 years
**Stage**	I–IIIb	I–IV	I–IV	N/A
**In-plane range (mm)**	0.721–0.977 (Avg. 0.976)	0.586–0.953 (Avg. 0.736)	0.617–0.977 (Avg. 0.785)	0.469–1.016 (Avg. 0. 598)
**Avg Slice thickness (mm)**	3.022	4.443	4.708	1.575

*NSCLC* non-small cell lung cancer, *TCIA* The Cancer Imaging Archive, *TCGA-KIRC* The Cancer Genome Atlas Kidney Renal Clear Cell Carcinoma, *CPTAC-GBM* Clinical Proteomic Tumor Analysis Consortium Glioblastoma Multiforme, *Avg* average, *M* males, *F* females

### Imaging differences at the whole organ level

Each of the three organs that we studied might have a distinct intensity distribution due to inherent differences in an organ, as well as the differences in an imaging modality. A region of interest (ROI) was specified for each organ as a whole, and intensity histogram and the derived parameters of the histogram were compared. The training set (lung), test set2 (kidney), and test set3 (brain) were used for the analysis. Test set1 (lung) was not analyzed as it was the same organ as that in the training set (lung). Detailed segmentation methods are described in the supplementary methods. Histogram shapes were visually compared. Means and medians of the ROIs were also compared using two-sample t-tests. Within CT imaging (i.e., lung and kidney), the histogram shape was directly compared. Across imaging modalities (CT [lung and kidney] and MRI [brain]), we normalized the histograms using z-scores. The z-score normalization was one way to compare CT that has the standard Hounsfield unit (HU) with MRI that has an arbitrary unit. We only applied histogram normalization to compare histograms from different organs and did not apply the normalization when extracting the radiomics features.

### ROI segmentation and feature extraction

On axial CT and MRI images, tumors were segmented using in-house semi-automated software by a single expert (H.Y.L) [22]. A total of 143 radiomics features were computed from the native volume space over the ROIs [8, 22, 23]. The features were computed using a combination of open-source (PyRadiomics) and in-house codes in MATLAB (MathWorks, Inc., Natick, USA) [24]. Features that were unavailable in the PyRadiomics were locally implemented. The features consisted of 19 histogram-based, 11 shape-based, three fractal-based, 18 sigmoid function-based, 29 texture-based, and 63 filter-based features. Eight shape-based features were extracted using a three-dimensional (3D) ROI, and three shape-based features were two-dimensional (2D) [25]. The 2D shape-feature used the center slice of the 3D ROI. Three fractal-based features were the fractal dimension, fractal signature dissimilarity, and lacunarity [26, 27]. Fractal dimension was computed using the box-counting, and fractal signature dissimilarity (FSD) was computed using the blanket method. Eighteen sigmoid function-based features were computed to quantify the tumor margin properties [25]. Twenty-nine texture-based features were computed using gray-level co-occurrence matrix (GLCM, 256 bins, *n* = 22), gray-level size zone matrix (GLSZM, 32 bins, *n =* 2), and neighborhood gray-tone difference matrix (NGTDM, *n* = 5) [20, 28–30]. GLCM features were computed in two ROI types using the whole ROI and sub-sampled ROI [23]. The filter-based features were computed using a 3D Laplacian of Gaussian (LoG) filter. The sigma values of the LoG filter were computed with σ in 0.5 voxel increments (range, 0.5–3.5) [25]. A detailed description of the features is given in the supplement.

### Selection of features

Radiomics analysis involved choosing a few features that can explain the intended target clinical variable well from many features. The feature selection was carried out in two steps in the training set. First, we chose stable features using the intra-class correlation (ICC) with IBM SPSS statistical software (IBM Corp., Armonk, USA). The stability of the 143 extracted features was assessed using the Reference Image Database to Evaluate Therapy Response (RIDER) dataset [31, 32]. We retained features with ICC values above 0.9 [33]. Second, we applied Cox - least absolute shrinkage selector operator (Cox-LASSO) to select a few features related to survival from the selected features obtained from the first step. The hyperparameter of LASSO (i.e., regularization strength) was optimized using cross-validation. The MATLAB **(**MathWorks, Inc., Natick, USA) and “glmnet” R package (R Foundation for Statistical Computing, Vienna, Austria) were used for the Cox-LASSO. The Cox-LASSO method was repeated 20 times, and only features that were selected more than ten times were chosen as the final selected features. In addition, we applied the Cox-LASSO feature selection to other datasets (test set1, 2, and 3) to seek common features between the training set and each test set and test alternative models based on different datasets. The procedure was applied with a reduced threshold (10 times out of 40) due to convergence issues.

### Construction of radiomics score model and survival analysis

**Fig. 1:**
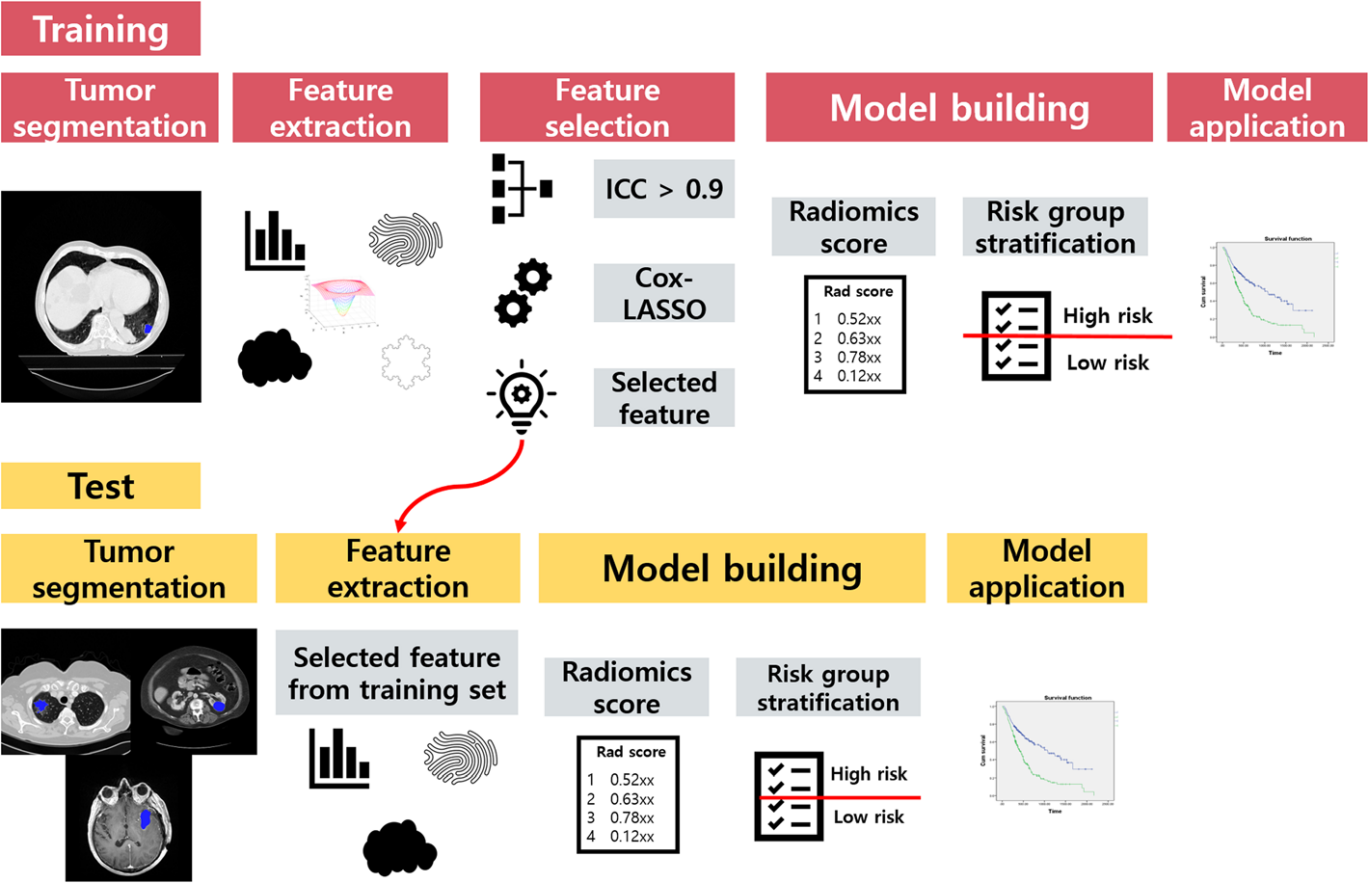
Overall scheme of the study

We built a radiomics score related to survival using the selected features. Regression coefficients from the Cox-LASSO were linearly combined with the feature values to yield a radiomics score. The radiomics score is a weighted (weights obtained from regression coefficients) sum of feature values [34, 35]. Each patient’s radiomics score was computed. Patients were stratified into low- and high-risk groups using the median radiomics score as the cutoff [36]. Kaplan-Meier (KM) survival analysis was performed, and a log-rank test was used to compare the two risk groups using SPSS. The procedure was applied to the training set and validated in the three test sets. We developed the radiomics score model consisting of the selected features from the lung dataset and applied the model using the same features but refitted the coefficients to test it on the brain and kidney cases. The overall scheme of the study is given in Fig. 1.

## Results

### Whole organ level imaging differences

**Fig. 2:**
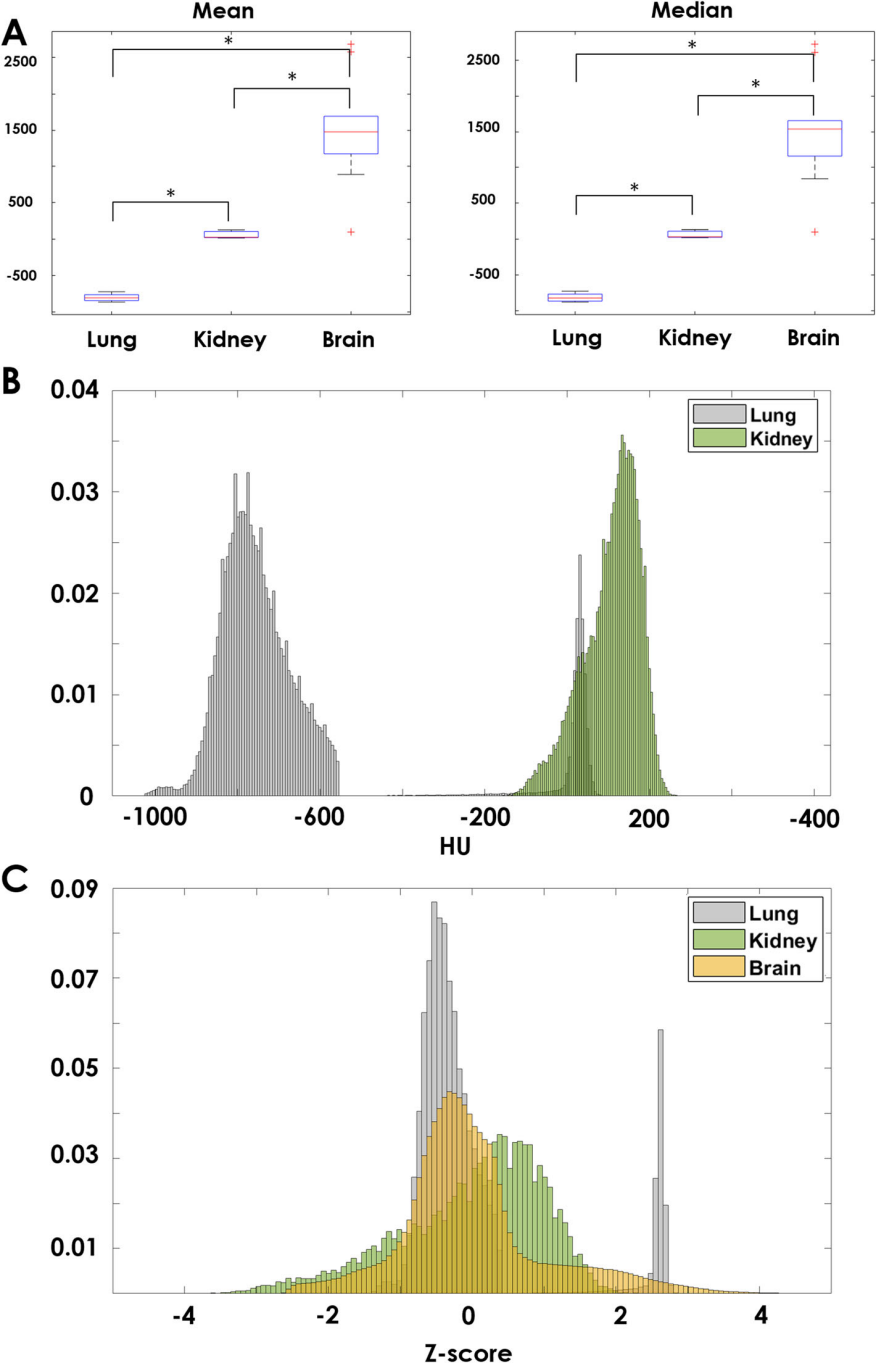
Imaging differences at the whole-organ level. **a** Box plots for mean and median; **b** comparison of histogram within CT for lung (gray color) and kidney (green color); and **c** comparison of normalized histogram across modalities for lung (gray color), kidney (green color), and brain (yellow color)

Based on the two-sample t-tests, mean values were significantly different between lung and kidney (*p* < 0.001, 3.43 × 10^− 9^), lung and brain (*p* = 1.41 × 10^− 8^), and kidney and brain (*p* = 9.53 × 10^− 6^). Similarly, median values were significantly different between lung and kidney (*p* = 7.07 × 10^− 19^), lung and brain (*p* = 1.22 × 10^− 5^), and kidney and brain (*p* = 1.74 × 10^− 8^). The box plots for mean and median values are shown in Fig. 2a.

The histogram shape was compared between CT images (lung and kidney) for a representative case (Fig. 2b). Intensity distribution did not overlap much between the two organs. Histogram was normalized to z-scores to make a comparison between CT and MRI modalities. All three distributions of the organs were centered at zero with distinct shapes for each organ (Fig. 2c).

### Selected features and radiomics score

In the first selection step using the ICC threshold, 97 features were selected as stable features. The selected 97 features were used in the second feature selection step using Cox-LASSO. Finally, 7 features were selected from the training set. The final selected features were as follows: histogram-based features (2.5 percentile and 97.5 percentile); shape-based features (sphericity, maximum 3D diameter, and roundness factor [2D]); and texture-based feature (an informational measure of correlation [sub-sampled GLCM] and size-zone variability). One of the features, 2.5 percentile, is a well-known lung-specific feature [37]. We computed another radiomics score with the 2.5 percentile feature removed using the six features, to see if removing lung-specific features would lead to better generalization to other organs (i.e., kidney and brain). The ensuing survival analysis was performed using two radiomics scores.

### Survival analysis using all the selected features

**Fig. 3:**
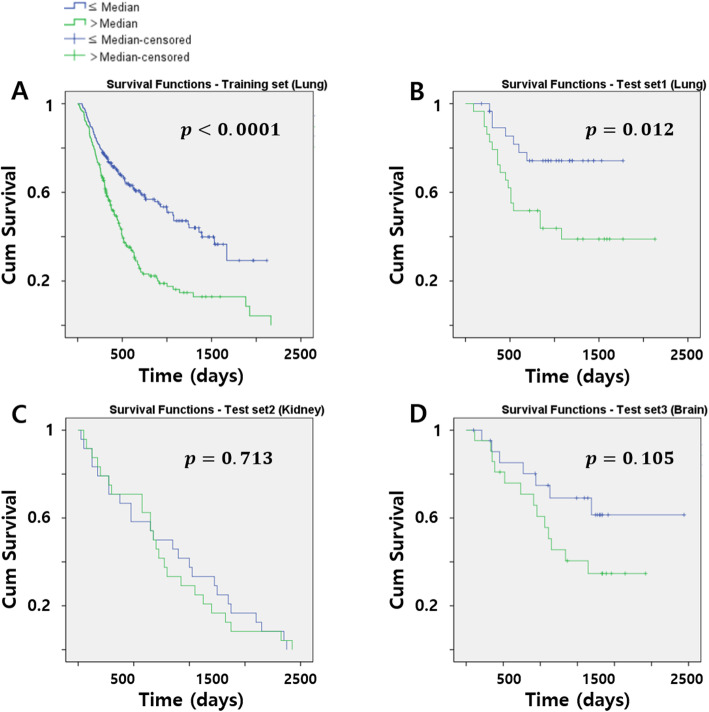
The Kaplan-Meier plots using the radiomics score of all the selected features of (**a**) training set (top left), (**b**) test set1 (top right), (**c**) test set2 (bottom left), and (**d**) test set3 (bottom right). The blue line is the low-risk group (≤ Median), and the green line is the high-risk group (> Median)

Survival analysis was performed, and patients were divided into high- and low-risk groups using the median radiomics score as the cut-off. The radiomics score was computed using all seven selected features from the training set. In the training set (lung), two risk groups were well-stratified using the log-rank test (*p* < 0.001) (Fig. 3a). There was a similar significant difference (*p* = 0.012) in survival between the two risk groups in the test set1 (lung) using the radiomics score computed from the test set1 (Fig. 3b). However, the results from test set2 and test set3 were different. The test set2 (kidney) did not show any significant differences in survival between the two risk groups, and the log-rank test was not statistically significant (*p* = 0.713) (Fig. 3c). In the test set3 (brain), differences in survival between the two risk groups were visually noticeable in the plot, but the difference was not statistically significant (*p* = 0.105) (Fig. 3d). In summary, the radiomics model trained from the lung data was only useful for stratifying survival in the other lung data, and not in other organs like the kidney and brain. Additional results of using kidney and brain datasets to build survival models were shown in the Supplement.

### Survival analysis without the lung-specific feature

**Fig. 4:**
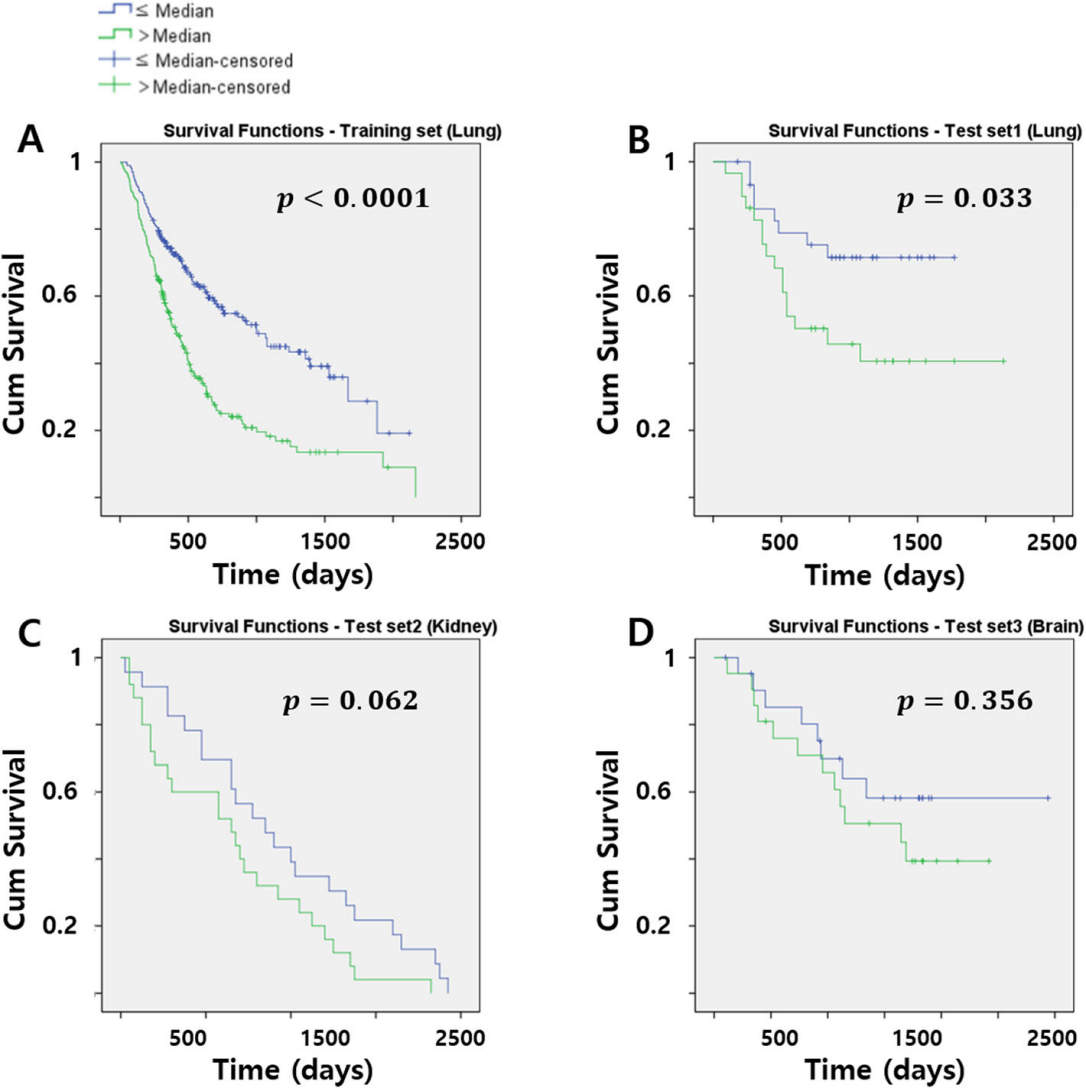
The Kaplan-Meier plots using radiomics score with the lung-specific feature removed of ((**a**) training set (top left), (**b**) test set1 (top right), (**c**) test set2 (bottom left), and (**d**) test set3 (bottom right). The blue line is the low-risk group (≤ Median), and the green line is the high-risk group (> Median)

The same survival analysis was performed using the radiomics score without the lung-specific 2.5 percentile feature. The results were largely similar to those using the radiomics score for all the features. In the training set (lung), the two risk groups were well-stratified using the log-rank test (*p* < 0.001) (Fig. 4a). There was a similar significant difference (*p* = 0.033) in the survival between the two risk groups in test set1 (lung) using the radiomics score computed from the test set1 (Fig. 4b). However, the results from test set2 and test set3 were different. The test set2 (kidney) showed visual differences in the survival plot but did not show any significant differences (*p* = 0.062) (Fig. 4c). In the test set3 (brain), differences in survival between the two risk groups were visually noticeable in the plot, but there was no statistical significance (*p* = 0.356) (Fig. 4d). In summary, the radiomics model trained from the lung data was only effective in stratifying survival in the other lung data, and not in other organs like the kidney and brain.

### Common features between the training set and test sets

We observed that the radiomics model trained from the lung data was only effective in stratifying survival in the other lung data, and not in other organs like the kidney and brain. This might imply that features reflecting survival could be different for different organs. Thus, we performed the same feature selection approach on all four datasets and compared the features selected among different datasets across three organs. In the test set1, variance, standard deviation, 50 percentile (histogram), homogeneity (sub-sampled GLCM), roundness factor (2D shape), and FSD (fractal) were selected. In test set2, energy, difference entropy (sub-sampled GLCM), and contrast (NGTDM) were selected. In the last test set3, informational measures of correlation (sub-sampled GLCM) and coarseness (NGTDM) were selected. The roundness factor (2D shape) was a common feature between the training set and test set1. No common features were found between the training set and test set2. The informational measure of correlation (sub-sampled GLCM) was a common feature between the training set and test set3. There were no common features between the training set and various test sets, but there was a category of features that was common. All datasets had at least one GLCM feature as the chosen feature denoting the importance of texture feature in radiomics. Full details are given in Table 2.

**Table 2:** Results of feature selection for each dataset

Dataset	Feature category	Feature name	Repetition times
**Training (7 features)**	Histogram-based	2.5 percentile	20
		97.5 percentile	20
	Shape-based (3D)	Sphericity	20
		Max 3D diameter	20
	Shape-based (2D)	**Roundness factor**	20
	Texture-based	**Informational Measure of Correlation, subsampled GLCM**	17
		Size zone variability	10
**Test set1** **(6 features)**	Histogram-based	Variance	20
		50 Percentile	20
	Texture-based	Homogeneity, subsampled GLCM	15
	Shape-based (2D)	**Roundness factor**	9
	Fractal-based	FSD	4
	Histogram-based	Standard deviation	1
**Test set2** **(3 features)**	Texture-based	Energy, subsampled GLCM	1
		Contrast, NGTDM	1
		Difference entropy, subsampled GLCM	1
**Test set3** **(2 features)**	Texture-based	**Informational Measure of Correlation, subsampled GLCM**	**19**
		Coarseness, NGTDM	3

Bold font indicates the features commonly selected between the training set and given test sets. *2D* two-dimensional, *3D* three-dimensional, *GLCM* gray-level co-occurrence matrix, *FSD* fractal signature dissimilarity, *NGTDM* neighborhood gray-tone difference matrix

## Discussion

We confirmed that there were obvious differences in imaging at the organ level for the three organs by comparing their intensity histograms. The radiomics score and the associated features derived from the training set (lung) were effective for the test set1 (the other lung) in stratifying survival, but not for the kidney and brain (test set2 and set3). A similar trend was observed even when we removed the lung-specific feature (2.5 percentile) from the radiomics score.

Many radiomics studies have discovered features related to the tumor microenvironment and the associated macroscale features. In particular, features in the texture category have been commonly selected for various radiomics studies. In one study, GLCM-based features contributed to the discovery of radiomics signatures predicting immune microenvironment and patient outcomes [15]. Texture-based features were identified as important features to predict immune responses [19]. Texture-based features, unlike the histogram and shape-based features, incorporate the neighborhood information and hence are better suited to describe the tumor microenvironment.

We explored whether features trained in a specific organ can be applied to other organs. In Fig. 2, we confirmed that different organs have distinct differences in intensity distribution. Figure 2b shows that a lung CT scan is quite different from that of the kidney due to the unique environment of the lungs, which includes a large proportion of air. This distinct information for each organ makes finding a universal feature set difficult across different organs. The features derived from the training set (lung) were not useful in stratifying survival in the kidney and brain (test set2 and set3) datasets. To correct for the differences among organs, additional experiments to remove the feature related to air components (2.5 percentile) were performed. This was to test whether removing the lung-specific feature could lead to better generalizability to the other two organs. However, the results were somewhat similar. Despite the lung-specific feature removed, the features derived from the training data could not be applied to the other two organs. This observation indicated that radiomics features of a given organ from a tumor ROI reflected a specific tumor microenvironment and the associated macroscale features. Therefore, applying the radiomics feature derived from one organ to other organs requires careful consideration of specific properties of the target organ.

Both training set and test set1 were from lung-cancer databases, but there was only one common feature selected (Table 2, roundness factor). This might imply that circularity is more important in lung cancer compared to other organs. The data of the training set came from various types of lung cancer patients: large cell, squamous cell carcinoma, and adenocarcinoma. However, the entire data of test set1 were derived from patients with adenocarcinoma. Thus, this difference in subtype composition could have led to different features being selected. Between the training set and test set3, one common feature was selected, the informational measure of correlation. This confirmed that lung cancer and glioblastoma were both associated with the secondary measure of homogeneity.

To the best of our knowledge, no radiomics study has attempted to find common features in different organs systematically. Some studies explored the concept of universal radiomics features, but it was their secondary objective, not the main one. One study adopted a procedure similar to ours. The study built radiomics models using lung cancer data and applied them to H&N cancer data. They showed that the same radiomics model was effective for predicting survival in two organs, but the results could be invalid because their feature selection approach was biased [20]. Another study attempted to explore the feasibility of applying the same set of radiomics features [21]. However, the goal was not to find common features but to compare the performance of 2D and 3D radiomics in intrahepatic cholangiocarcinoma, osteosarcoma, and pancreatic neuroendocrine tumors.

Although common features were elusive, we reconfirmed that texture features could be a common category of features for the three organs. This finding is largely in line with many radiomics studies emphasizing the importance of texture features to capture tumor heterogeneity. Active research is ongoing to propose new texture features, some of which could be applied to many organs.

Recently, we witnessed increased adoption of deep learning (DL) in imaging analysis that led to improved performances in many organs [38–40]. DL approaches use more features (even in tens of thousands) than radiomics approaches (hundreds to thousands) in a data-driven fashion. The intermediate layers of the DL network are commonly used for data-driven feature representation. As there are more features to choose from, there could be a better chance of finding common features between different organs. DL approaches are inherently multi-scale, and thus common features might be found in small-scale low-level features.

Our study has some limitations. The sample size of the test sets was rather small (*n* < 100); thus, further studies using larger samples are necessary to validate the results of our study. We explored radiomics models for survival analysis and did not consider other clinical outcomes, such as tumor grading, that need to be explored in the future. Our results were limited to three organs (lung, kidney, and brain) and two imaging modalities (CT and MRI). Therefore, future studies may extend this approach to more organs and modalities. Our model from the training set had three subtypes of NSCLC and thus might not have the transfer capability to one specific subtype of NSCLC. Exploring the subtype-specific transferability is an important future research direction. Finally, there is no agreed standard method to extract and select radiomics features, which makes tight controlling of the experiments difficult. Although our method is a widely used one [23, 36, 41], our results were specific to the adopted methods and thus should be interpreted with care.

## Conclusion

Overall, we suggest that radiomics score models for survival were mostly specific for a given organ. This was confirmed by the absence of any common features being identified between the training set and various test sets. However, we noticed that one category of features (texture category) was common in all three organs. In sum, applying radiomics score models to other organs would require careful consideration of organ-specific properties. Still, caution should be taken when constructing models with radiomics features because we cannot exclude the possibility to construct a generally applicable model for various organs given an optimal model.

## Supplementary Information

**Additional file 1: Supplementary Table 1.** Definition of extracted radiomics features. **Supplementary Methods**. Patients and imaging datasets. Imaging differences at the organ level. **Supplementary Results. Fig. S1.** The Kaplan-Meier plots using the radiomics score based on test set2 (kidney). **Fig. S2.** The Kaplan-Meier plots using the radiomics score based on test set2 (brain).

## Data Availability

The data and material are available through one of the corresponding authors (Dr. Ho Yun Lee).

## Abbreviations

NSCLC: Non-small cell lung cancer
HU: Hounsfield unit
LoG: Laplacian of Gaussian
GLCM: Gray-level co-occurrence matrix
GLSZM: Gray-level size zone matrix
NGTDM: Neighborhood gray-tone difference matrix
FSD: Fractal signature dissimilarity
Cox-LASSO: Cox - least absolute shrinkage selector operator
ICC: Intra-class correlation
KM: Kaplan-Meier
