# Supplementary material for: Are radiomics features universally applicable to different organs?
Source: Cancer Imaging. 2021 Apr 7;21:31. doi: 10.1186/s40644-021-00400-y (PMC8028225; doi:10.1186/s40644-021-00400-y)
Supplement: Supplementary file 1 — Additional file 1: Supplementary Table 1. Definition of extracted radiomics features. Supplementary Methods. Patients and imaging datasets. Imaging differences at the organ level. Supplementary Results. Fig. S1. The Kaplan-Meier plots using the radiomics score based on test set2 (kidney). Fig. S2. The Kaplan-Meier plots using the radiomics score based on test set2 (brain). [file 40644_2021_400_MOESM1_ESM.docx]

**Supplementary Material for:**

**Are radiomics features universally applicable to different organs?**

**Supplementary table**

**TABLE 1** Definition of extracted radiomics features

|  | **Parameter** | **Formula** | **Description** | **IBSI** [1] **compliant** |
| --- | --- | --- | --- | --- |
| **Histogram-based features (4096 bins)** [2] | Max, Min | $\mathrm{Max}=Max(X(i))$ or $\mathrm{Min}=Min(X(i))$  Where $X$ denotes the 3d image matrix with $N$ voxel. | Measures maximum or minimum intensity value of a histogram | O |
|  | Median | $Median=\frac{X(i)}{2}$  Where $X$ denote the 3d image matrix | Measures median intensity value of a histogram | O |
|  | Mean | $Mean=\frac{1}{N}\sum_{i}^{N} X(i)$  Where $X$ denote the 3d image matrix with $N$ voxel. | Measures mean intensity value of a histogram | O |
|  | Variance | $Variance=\frac{1}{N-1}\sum_{i=1}^{N} \left( X\left( i \right)-\bar{x} \right)^{2}$ | Measures squared distances of each value of a histogram from the mean | O |
|  | Standard deviation | $Std=\left( \frac{1}{N-1}\sum_{i=1}^{N} \left( X\left( i \right)-\bar{x} \right)^{2} \right)^{1/2}$  Where $X$ denote the 3d image matrix with $N$ voxel. | Measures amount of variation of a histogram. | O |
|  | Energy | $Energy=\sum_{i}^{N} {X(i)}^{2}$  Where $X$ denote the 3d image matrix with $N$ voxel. | Measures squared magnitude value of a histogram | O |
|  | Skewness | $Skewness=\frac{E{(x-\mu)}^{3}}{\sigma^{3}}$  Where $\mu$ is the mean of $x$, $\sigma$ is the standard deviation of $x$, $E$ is the expectation operator. | Measures asymmetry of a histogram. | O |
|  | Kurtosis | $Kurtosis=\frac{{E(x-\mu)}^{4}}{\sigma^{4}}$  Where $\mu$ is the mean of$x$, $\sigma$ is the standard deviation of $x$, $E$ is the expectation operator. | Measures “peakedeness” of a histogram (flatness of histogram) | O |
|  | Root mean square (RMS) | $RMS=\sqrt{\frac{1}{N}\sum_{n=1}^{N} \left\vert X_{n} \right\vert^{2}}$  Where $X$ denote the 3d image matrix with $N$ voxel. | Measures the square-root of the mean of the squares of the values of the histogram. This feature is another measure of the magnitude of a histogram | O |
|  | Inter quartile range | $IQR=Q_{3}-Q_{1}$  Where $Q_{3}$ denote the 3^rd^ quartile of histogram, $Q_{1}$ denote the 1^st^ quartile of histogram | Measures of variability, based on dividing a histogram into quartiles | O |
|  | Range | $Range=range(X\left( i \right))$ | Measures difference between the highest and lowest voxel values of a histogram | O |
|  | Percentile | $Percentile=\left( \frac{n^{th} percentile}{100} \right) X(i)$ | Measures intensity value at the 2.5^th^, 25^th^ ,50^th^ ,75^th^, and 97.5^th^ percentile on histogram | O |
|  | Entropy | $\mathrm{Entropy}=-\sum_{i=1}^{N_{l}} P(i)\log_{2} P(i)$  Where $P$ denotes the first-order histogram with $N_{l}$ discrete intensity levels. | Measures irregularity of a histogram. | O |
|  | Uniformity | $Uniformity=\sum_{i=1}^{N_{l}} {P(i)}^{2}$  Where $P$ denotes the first-order histogram with $N_{l}$ discrete intensity levels. | Measures uniformity of a histogram. | O |
|  | Mean value of positive pixels (MPP) | $\mathrm{MPP}=\frac{1}{N_{+}}\sum_{i}^{N} X(i)$  Where $N_{+}$ denotes the total number of positive gray level pixels in $X\left( i \right)$ | Measures average positive histogram value. | O |
|  | Uniformity value of positive pixels (UPP) | $UPP=\sum_{i=1}^{N_{l}} \left\vert P(i) \right\vert^{2}$  Where $P$ denotes the first-order histogram with $N_{l}$ discrete intensity levels. | Measures uniformity of positive histogram value. | O |
| **Shape- and size-based features** [2,3] | Compactness | $Compactness=\frac{V}{\sqrt{\pi}A^{\frac{3}{2}}}$  Where$V$ denotes the volume, and $A$ denotes the surface area of the volume of interest (VOI) | Quantifies how close an object is to the smoothest shape, the circle | O |
|  | Surface area | $SA=\sum_{i=1}^{N} \frac{1}{2}\left\vert a_{i}b_{i}\times a_{i}c_{i} \right\vert$  Where $N$ is the total number of triangles (coved surface area), and $a,b, c$ are edge vectors | The surface area of the ROI | O |
|  | Convexity | $\mathrm{Convexity}=\frac{V}{V^{'}}$  Where $V$ denotes tumor volume, and $V^{'}$ denotes convex hull volume | Measures ratio of the ROI volume contained within the tumor to the calculated convex hull volume | O |
|  | Sphericity | $Sphericity=\frac{\pi^{\frac{1}{3}}\times{(6V)}^{\frac{2}{3}}}{A}$  Where $A$ denotes area, and $V$ denotes tumor volume | Measures of the roundness of the ROI | O |
|  | Spherical disproportion | $Spherical disproportion=\frac{A}{4\pi R^{2}}$  Where $R$ is the radius of a sphere with the same volume as the tumor | The ratio of the surface area of the ROI to the surface area of a sphere with the same volume as the ROI | O |
|  | Maximum 3D diameter | See description in the next column | Measures of the maximum 3D ROI diameter. It is measured as the largest pairwise Euclidean distance, between surface voxels of the ROI | O |
|  | Surface to volume ratio (SVR) | $SVR=\frac{A}{V}$  Where $A$ is area, and $V$ is volume | Surface to volume ratio in ROI | O |
|  | Volume | $Volume=R*number of voxels$  Where $R$ denote the 3d image resolution | Volume of tumor (ROI) | O |
|  | Mass | $Mass=V*D$  Where $V$ denote the tumor volume, $D$ denote the tumor density | Mass of tumor (ROI) |  |
|  | Density | $Density=\frac{M}{V}$  Where $V$ denote the tumor volume, $M$ denote the tumor mass | Density of tumor (ROI) |  |
|  | Roundness factor (2D) | $Roundness factor=\frac{4\pi\cdot Area}{{Perimeter}^{2}}$ | Measure of circularity of a ROI | O |
|  | Eccentricity (2D) | $Eccentricity=c/a$  Where $c$ is the distance from the center to a focus and $a$ is the distance from that focus to a vertex | Measure of how the tumor shape is close to  the circle | O |
|  | Solidity (2D) | $Solidity= \frac{Area}{Convex area}$ | Measure of convexity of a ROI on the 2D image | O |
| **GLCM-based features (256 bins)** [2] | Auto correlation | $Autocorrelation =\sum_{i=1}^{N_{g}} \sum_{j=1}^{N_{g}} ij\boldsymbol{P}(i,j)$ | Measures of the magnitude of the fineness and coarseness of texture | O |
|  | Cluster tendency | $Cluster tendency=\sum_{i=1}^{N_{g}} \sum_{j=1}^{N_{g}} \left[ i+j-\mu_{x} -\mu_{y} \right]^{2}\mathbf{P}(i,j)$ | Measures of the homogeneity of GLCM | O |
|  | Maximum probability | $Maximum probability=max\{P\left( i,j \right)\}$ | Measures maximum value of GLCM matrix | O |
|  | Contrast | $Contrast=\sum_{i=1}^{N_{g}} \sum_{j=1}^{N_{g}} \left\vert i-j \right\vert^{2}\mathbf{P}(i,j)$ | Measures of the local intensity variation of GLCM | O |
|  | Difference entropy | $Difference entropy=\sum_{i=0}^{N_{g}-1} \mathbf{P}_{x-y}(i)\log_{2} [P_{x-y}(i)]$ | Measures entropy of processed GLCM matrix Px-y | O |
|  | Dissimilarity | $Dissimilarity=\sum_{i=1}^{N_{g}} \sum_{j=1}^{N_{g}} \left\vert i-j \right\vert\mathbf{P}(i,j)$ | Measures difference of each element of the gray level | O |
|  | Energy | $Energy=\sum_{i=1}^{N_{g}} \sum_{j=1}^{N_{g}} \left[ \mathbf{P}\left( i,j \right) \right]^{2}$ | Measures of the homogeneity of GLCM | O |
|  | Entropy | $Entropy=-\sum_{i=1}^{N_{g}} \sum_{j=1}^{N_{g}} \mathbf{P}(i,j)\log_{2} [\mathbf{P}\left( i,j \right)]$ | Measures irregularity of gray level. | O |
|  | Homogeneity | $Homogeneity=\sum_{i=1}^{N_{g}} \sum_{j=1}^{N_{g}} \frac{\mathbf{P}\left( i,j \right)}{1+\left\vert i-j \right\vert}$ | Measures closeness of gray-level. | O |
|  | Informational measure of correlation | $IMC=HXY-\frac{HXY1}{max\{HX,HY\}}$ | Secondary measure of Homogeneity | O |
|  | Variance | $Variance=\sum_{i=1}^{N_{g}} \sum_{j=1}^{N_{g}} \left( i-\mu_{x} \right)^{2}P(i,j)$ | Measures dispersion of the parameter values around the mean of the combinations of reference and neighborhood pixels | O |
|  | Where $\mathbf{P}\left( i,j \right)$is the gray level co-occurrence matrix for ($\delta=1, \alpha=0)$,  $N_{g}$is the number of discrete intensity value in the image,  $N$ is the number of voxels in the ROI,  $\mu$ is the mean of $\mathbf{P}\left( i,j \right),$  $p_{x}\left( i \right)=\sum_{j=1}^{N_{g}} \mathbf{P}(i,j)$ is the marginal row probabilities,  $p_{y}\left( i \right)=\sum_{i=1}^{N_{g}} \mathbf{P}(i,j)$ is the marginal column probabilities,  $\mu_{x}$ is the expected value of marginal row probability,  $\mu_{y}$ is the expected value of marginal column probability,  $\sigma_{x}$ is the standard deviation of $p_{x}$,  $\sigma_{y}$ is the standard deviation of $p_{y}$,  $p_{x+y}\left( k \right)=\sum_{i=1}^{N_{g}} \sum_{j=1}^{N_{g}} \mathbf{P}\left( i,j \right) , i+j=k, k=2,3,\ldots,2N_{g}$,  $p_{x-y}\left( k \right)=\sum_{i=1}^{N_{g}} \sum_{j=1}^{N_{g}} \mathbf{P}\left( i,j \right) ,\left\vert i-j \right\vert=k, k=0,1,\ldots,N_{g}-1$,  $HX=-\sum_{i=1}^{N_{g}} \mathbf{P}_{x}(i)\log_{2} \left[ p_{x}(i) \right]$ is the entropy of $\mathbf{P}_{x}$,  $HY=-\sum_{i=1}^{N_{g}} \mathbf{P}_{y}(i)\log_{2} \left[ p_{y}(i) \right]$ is the entropy of $\mathbf{P}_{y}$,  $HXY=-\sum_{i=1}^{N_{g}} \sum_{j=1}^{N_{g}} \mathbf{P}\left( i,j \right)\log_{2} \left[ \mathbf{P}(i,j) \right]$is the entropy of $\mathbf{P}\left( i,j \right)$  $HXY1=-\sum_{i=1}^{N_{g}} \sum_{j=1}^{N_{g}} \mathbf{P}\left( i,j \right)\log(p_{x}\left( i \right)p_{y}\left( j \right))$ | | | |
| **GLSZM-based features (32 bins)** [4] | Size-zone variability | $Size zone variability=\frac{1}{\Theta}{\sum_{m=1}^{M} \left[ \sum_{n=1}^{N} \boldsymbol{P}\left( m,n \right) \right]}^{2}$ | Variability in the size of ROI | O |
|  | Intensity variability | $Intensity variability=\frac{1}{\Theta}{\sum_{n=1}^{N} \left[ \sum_{m=1}^{M} \boldsymbol{P}\left( m,n \right) \right]}^{2}$ | Variability in the intensity of ROI | O |
|  | Where $\boldsymbol{P}\left( m,n \right)$ is the intensity size zone matrix  $\Theta$ represents the number of homogeneous areas in tumor,  $M$ is the number of distinct intensity values,  $N$ is the size of homogeneous area in the matrix $\boldsymbol{P}\left( m,n \right)$ | | | |
| **NGTDM-based features** [5,6] | Busyness | $Busyness=\sum_{i=1}^{L} p_{i}s(i)/\sum_{i=1}^{L} \sum_{j=1}^{L} (ip_{i}-jp_{j})$ | Measure of spatial rate of gray-level change | O |
|  | Coarseness | $\mathrm{Coarseness}=\left[ \sum_{i=1}^{L} p_{i}s(i) \right]^{-1}$ | Measure of edge density | O |
|  | Complexity | $\mathrm{Complexity}=\sum_{i=1}^{L} \sum_{j=1}^{L} \left\{ \left( \left\vert i-j \right\vert\right)/(n^{2}\left( p_{i}+p_{j} \right)) \right\}\left\{ P_{i}s\left( i \right)+p_{j}s(j) \right\}$ | Measure of the amount of information in a ROI (gray-level intensities, number of sharp edges) | O |
|  | Contrast | $\mathrm{Contrast}=\frac{1}{N_{g}(N_{g}-1)}\sum_{i=1}^{L} \sum_{j=1}^{L} {p_{i}p_{j}\left( i-j \right)}^{2}\cdot\frac{1}{n^{2}}\sum_{i=1}^{L} s(i)$ | Measure of local variations and spread of matrix values | O |
|  | Strength | $\mathrm{Strength}=\sum_{i=1}^{L} \sum_{j=1}^{L} ({p_{i}+p_{j})\left( i-j \right)}^{2}/\sum_{i=1}^{L} s(i)$ |  | O |
|  | Where $p_{i}$ is the probability of occurrence of gray level value, $s(i)$ is the NGTDM, $N_{g}$ is the total number of different gray levels in the ROI, $L$ is the number of possible gray levels | | | |
| **Filter-based features** [3]  **(LoG)** | Mean | $Mean=\frac{1}{N}\sum_{i}^{N} G(i)$  Where $G$ denote the filtered 3d image matrix with $N$ voxel. | Measurement of mean of ROI image processed by LoG filter | O |
|  | Max | $\mathrm{Max}=Max(G(i))$  Where $G$ denotes the filtered 3d image matrix with $N$ voxel. | Measurement of max intensity value of ROI image processed by LoG filter | O |
|  | Min | $\mathrm{Min}=Min(G(i))$  Where $G$ denotes the filtered 3d image matrix with $N$ voxel. | Measurement of minimum intensity value of ROI image processed by LoG filter | O |
|  | Median | $Median=\frac{G(i)}{2}$  Where $G$ denote the filtered 3d image matrix | Measurement of median intensity value of ROI image processed by LoG filter | O |
|  | Standard deviation (Std) | $Std=\left( \frac{1}{N-1}\sum_{i=1}^{N} \left( G\left( i \right)-\bar{G} \right)^{2} \right)^{1/2}$  Where $G$ denote the filtered 3d image matrix with $N$ voxel. | Measurement of standard deviation of ROI image processed by LoG filter | O |
|  | Skewness | $Skewness=\frac{E{(G-\mu)}^{3}}{\sigma^{3}}$  Where $\mu$ is the mean of $G$, $\sigma$ is the standard deviation of $G$, $E$ is the expectation operator. | Measurement of skewness of ROI image processed by LoG filter | O |
|  | Kurtosis | $Kurtosis=\frac{{E(G-\mu)}^{4}}{\sigma^{4}}$  Where $\mu$ is the mean of$G$ $\sigma$ is the standard deviation of $G$, $E$ is the expectation operator. | Measurement of kurtosis of ROI image processed by LoG filter | O |
|  | Uniformity | $Uniformity=\sum_{i=1}^{N_{l}} {P(i)}^{2}$  Where $P$ denotes the first-order histogram with $N_{l}$ discrete intensity levels. | Measurement of uniformity of ROI image processed by LoG filter | O |
|  | Entropy | $\mathrm{Entropy}=-\sum_{i=1}^{N_{l}} P(i)\log_{2} P(i)$  Where $P$ denotes the first-order histogram with $N_{l}$ discrete intensity levels. | Measurement of entropy of ROI image processed by LoG filter | O |
|  | $G\left( x,y,z,\sigma\right)=I\left( x,y,z \right)*\frac{1}{{\sigma\left( \sqrt{2\pi} \right)}^{3}}e^{-\frac{x^{2}+y^{2}+z^{2}}{2\sigma^{2}}}$  $\sigma=0.5-3.5, 0.5 increments$, where I(x,y,z) is the image, and * denote convolution | | | |
| **Fractal-based features** [7,8] | Lacunarity (Box-counting method) | See description in the next column | Measure of the texture or distribution of gaps within an image | X |
|  | Dimension  (Box-counting method) | $Fractal dimension=\lim_{r\to0} \frac{\log(N_{r})}{\log(1/r)}$  Where $N_{r}$ is the number of voxels, and $r$ is the each of different side lengths | Fractal dimension quantifies morphological complexity and provides information on the self-similarity properties | X |
|  | Fractal signature dissimilarity  (Blanket method) | See description in the next column | Measure of tumor heterogeneity infromation | X |
| **Sigmoid function-based features** [3] | Amplitude mean | See description in the next column | Mean of the amplitude values of all sampling lines | X |
|  | Amplitude standard deviation |  | Standard deviation of the amplitue values of all sampling lines | X |
|  | Slope mean |  | Mean of the slope values of all sampling lines | X |
|  | Slope standard deviation |  | Standard deviation of the slope values of all sampling lines | X |
|  | Offset mean |  | Mean of the offset values of all sampling lines | X |
|  | Offset standard deviation |  | Standard deviation of the offset values of all sampling lines | X |
|  | $\mathrm{Sigmoid}\left( x \right)=\frac{A}{e^{\boldsymbol{B\cdot x}}+1}+C$  Where $A$ is the amplitude, $B$ is the slopeof the curve, and $C$ is the offset of the curve | | | |

GLSZM: gray-level size zone matrix; GLCM: gray-level co-occurrence matrix; NGTDM: Neighborhood gray tone difference matrix; LoG: Laplacian of Gaussian

**Supplementary Methods**

*Patients and imaging datasets*

- Dataset 1 (training set, lung, computed tomography [CT])

This training dataset was an NSCLC dataset used by Aerts et al [2,9]. We used CT images from the NSCLC radiomics data set obtained from The Cancer Imaging Archive (TCIA). The training set included 422 NSCLC patients (290 male and 132 female). Of the total 422 CT images, we used 401 for the analysis. The 401 cases were further divided into adenocarcinoma (n=49), large cell (n=110), squamous cell carcinoma (n=144), not otherwise specified (n=58), and unknown (n=40) types. We excluded 21 images because some radiomics features were not computed in them. The mean age of the patients was 68.1 years, and they belonged to stages I–IIIb. All patients received an FDG PET-CT scan for radiotherapy treatment planning in a radiotherapy position on a dedicated PET-CT simulator with both arms above the head before treatment. CT scans were obtained using the Siemens Biograph scanner and the CT scan was a spiral CT scan of the whole thorax with intravenous contrast. The CT image parameters of the training set were as follows: tube voltage, 120-140 kV; tube current-exposure time product, 40-1200 mAs;[10] mean in-plane resolution, 0.976 ± 0.016 mm (range, 0.721–0.977 mm); and mean slice thickness, 3.022 ± 0.256 mm. The values following the mean are standard deviation (SD) values.

- Dataset 2 (test set1, lung, CT)

The first test dataset was the TCIA lung CT diagnosis dataset provided by the H. Lee Moffitt Cancer Center [9,11]. There were 61 patients with lung cancer (31 males and 30 females; 41 patients of age ≥ 65 years). All patients in test set1 were diagnosed with adenocarcinoma of stages I–IV and underwent surgical resection and had corresponding pre-surgery diagnostic CTs obtained within 60 days of the diagnosis. Two patients were excluded because some of their radiomics features were not computed, and therefore, contrast-enhanced CT images of 59 patients were used for the analysis. The CT image parameters of the test set 1 were as follows: tube voltage, 120-140 kV; mean in-plane resolution 0.736 ± 0.09 mm (range, 0.586–0.953 mm); and average slice thickness, 4.443 ± 0.794 mm.

- Dataset 3 (test set2, kidney, CT)

The second test set was The Cancer Genome Atlas Kidney Renal Clear Cell Carcinoma (TCGA-KIRC) dataset [9,12]. Of the total 267 renal clear cell carcinoma patients, we randomly selected 48 patients (29 males and 19 females; mean age, 65.3 years; stages I–IV) and considered their contrast-enhanced CT imaging data. The CT image parameters were as follows: tube voltage, 120 kV; tube current-exposure time product, 265-400 mAs;[13] mean in-plane resolution, 0.785 ± 0.09 mm (range, 0.617–0.977 mm); and average slice thickness, 4.708 ± 1.606 mm.

- Dataset 4 (test set3, brain, MRI)

The third test set was the Clinical Proteomic Tumor Analysis Consortium Glioblastoma Multiforme (CPTAC-GBM) dataset [9,14]. Of the total 59 glioblastoma multiforme patients, we analyzed contrast-enhanced T1w-MRI, not CT, data from 43 patients (31 males and 12 females; mean age 63 years; stages I–IV). We excluded 16 patients due to a lack of survival information. The mean in-plane resolution was 0.598 ± 0.187 mm (range, 0.469–1.016 mm), and the average slice thickness was 1.575 ± 1.567 mm.

*Imaging differences at the organ level*

The whole lung was automatically segmented using the National Institutes of Health Center for Infectious Disease Imaging lung segmentation tool [15]. The kidney was segmented using an in-house semi-automated software, by a single expert (H.Y.L) [16]. The brain was automatically segmented using the FMRIB software library [17–19].

**Supplementary Results**

Using test set2 (kidney) as the training set, we chose three features GLCM difference entropy (sub-sampled), GLCM energy (subsampled), and NGTDM contrast. They were further used to build the Cox-LASSO model. The survival analysis using the model developed from the kidney showed that the risk group could be well stratified in the lung datasets (i.e., the original training set and test set1), but failed in stratification in the kidney and brain tumor datasets (i.e., test set2 and set3) as shown in Figure S1.


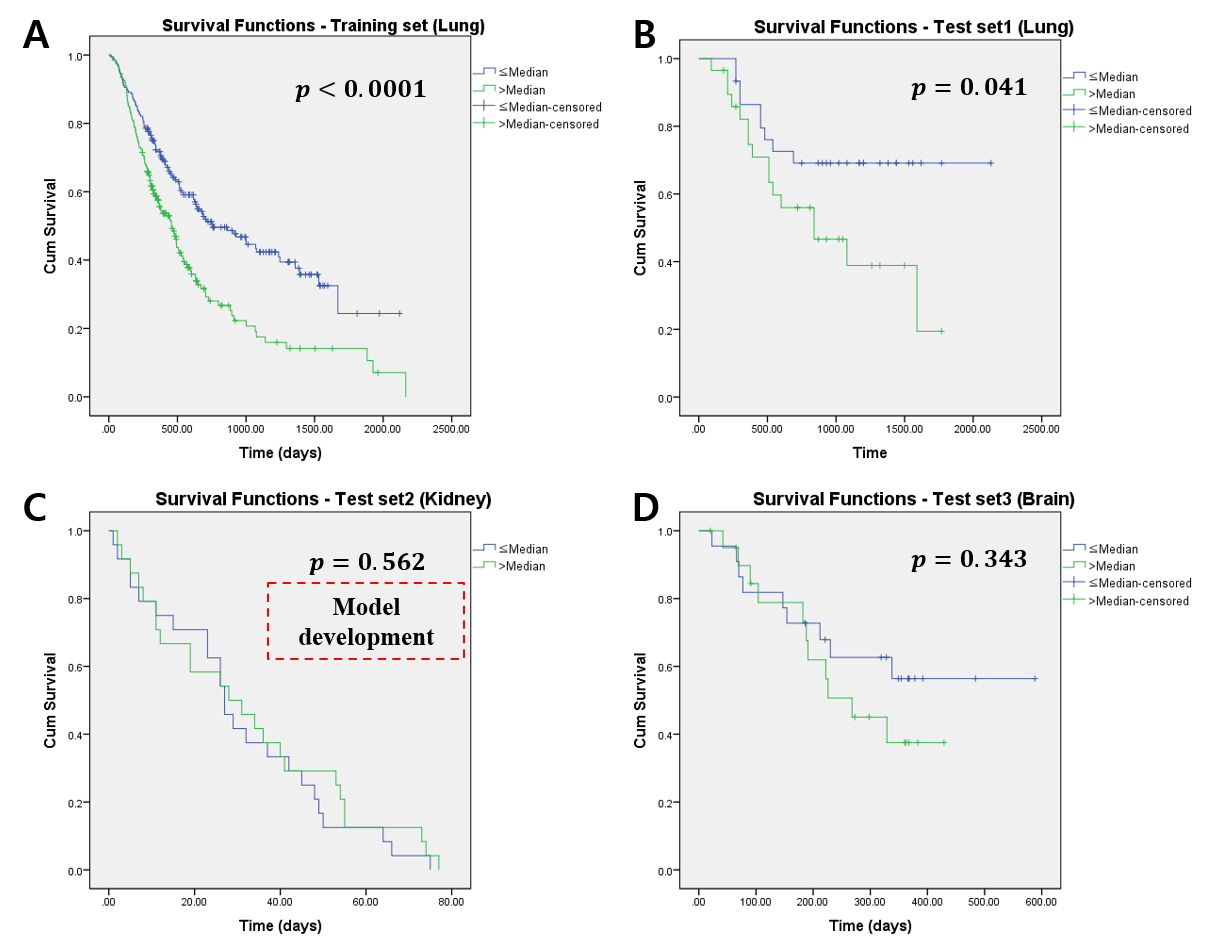


Figure S1. The Kaplan-Meier plots using the radiomics score based on test set2 (kidney). (A) training set (top left), (B) test set1 (top right), (C) test set2 (bottom left, model development), and (D) test set3 (bottom right). The blue line is the low-risk group (≤ Median), and the green line is the high-risk group (> Median). (A) and (B) showed significant group differences, but (C) and (D) showed no significant group differences.

Using test set3 (brain) as the training set, we chose informational measure of correlation and NGTDM coarseness. The survival analysis using the model developed from the brain showed that the risk group could be well stratified in one lung dataset (i.e., original training set) but failed in stratification other datasets (test set 1 of lung, test set2, and test set3) as shown in Figure S2.


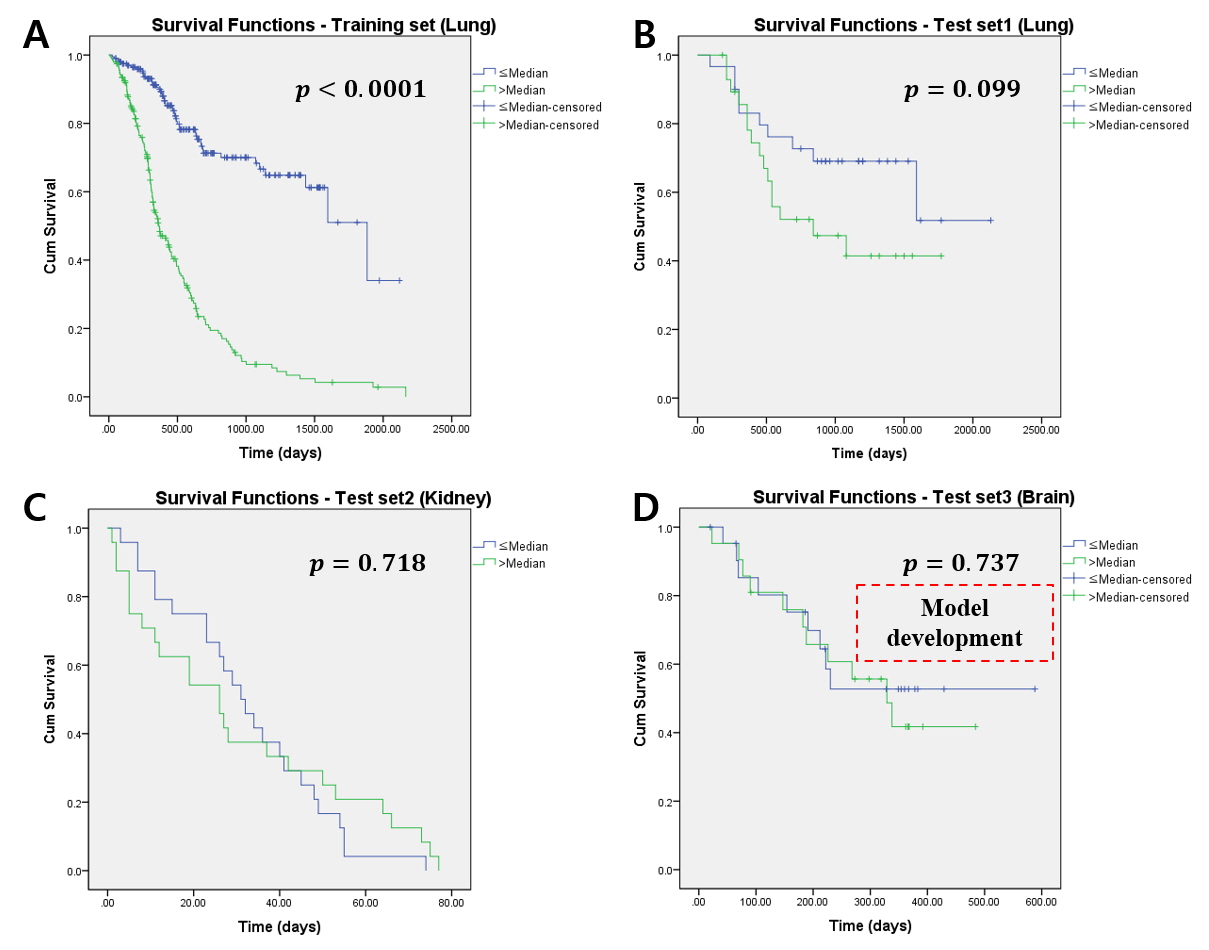


Figure S2. The Kaplan-Meier plots using the radiomics score based on test set2 (brain). (A) training set (top left), (B) test set1 (top right), (C) test set2 (bottom left), and (D) test set3 (bottom right, model development). The blue line is the low-risk group (≤ Median), and the green line is the high-risk group (> Median). Only (A) showed significant group differences. (B), (C) and (D) showed no significant group differences.

**Supplementary Reference**

1. Zwanenburg A, Vallières M, Abdalah MA, Aerts HJWL, Andrearczyk V, Apte A, et al. The Image Biomarker Standardization Initiative: Standardized Quantitative Radiomics for High-Throughput Image-based Phenotyping. Radiology. 2020;191145.

2. Aerts HJWL, Velazquez ER, Leijenaar RT, Parmar C, Grossmann P, Carvalho S, et al. Decoding tumour phenotype by noninvasive imaging using a quantitative radiomics approach. Nat Commun. 2014;5:4006.

3. Aerts HJWL, Grossmann P, Tan Y, Oxnard GG, Rizvi N, Schwartz LH, et al. Defining a Radiomic Response Phenotype: A Pilot Study using targeted therapy in NSCLC. Sci Rep. Nature Publishing Group; 2016;6.

4. Chong Y, Kim JH, Lee HY, Ahn YC, Lee KS, Ahn MJ, et al. Quantitative CT variables enabling response prediction in neoadjuvant therapy with EGFR-TKIs: Are they different from those in neoadjuvant concurrent chemoradiotherapy? PLoS One. 2014;9:1–8.

5. Niu L, Qian M, Yang W, Meng L, Xiao Y, Wong KKL, et al. Surface Roughness Detection of Arteries via Texture Analysis of Ultrasound Images for Early Diagnosis of Atherosclerosis. PLoS One. 2013;8.

6. Davnall F, Yip CSP, Ljungqvist G, Selmi M, Ng F, Sanghera B, et al. Assessment of tumor heterogeneity: An emerging imaging tool for clinical practice? Insights Imaging. 2012;3:573–89.

7. Lennon FE, Cianci GC, Cipriani NA, Hensing T a, Zhang HJ, Chen C-T, et al. Lung cancer—a fractal viewpoint. Nat Rev Clin Oncol. Nature Publishing Group; 2015;12:664–75.

8. Wang C, Subashi E, Yin FF, Chang Z. Dynamic fractal signature dissimilarity analysis for therapeutic response assessment using dynamic contrast-enhanced MRI. Med Phys. 2016;43:1335–47.

9. Clark K, Vendt B, Smith K, Freymann J, Kirby J, Koppel P, et al. The cancer imaging archive (TCIA): Maintaining and operating a public information repository. J Digit Imaging. 2013;26:1045–57.

10. Welch ML, McIntosh C, Haibe-Kains B, Milosevic MF, Wee L, Dekker A, et al. Vulnerabilities of radiomic signature development: The need for safeguards. Radiother Oncol [Internet]. The Author(s); 2019;130:2–9. Available from: https://doi.org/10.1016/j.radonc.2018.10.027

11. Grove O, Berglund AE, Schabath MB, Aerts HJWL, Dekker A, Wang H, et al. Quantitative computed tomographic descriptors associate tumor shape complexity and intratumor heterogeneity with prognosis in lung adenocarcinoma. PLoS One. 2015;10:1–14.

12. Akin, O., Elnajjar, P., Heller, M., Jarosz, R., Erickson, B. J., Kirk, S.,Filippini J. Radiology Data from The Cancer Genome Atlas Kidney Renal Clear Cell Carcinoma [TCGA-KIRC] collection. Cancer Imaging Arch. 2016;

13. Kocak B, Durmaz ES, Kaya OK, Ates E, Kilickesmez O. Reliability of single-slice–based 2D CT texture analysis of renal masses: Influence of intra- And interobserver manual segmentation variability on radiomic feature reproducibility. Am J Roentgenol. 2019;213:377–83.

14. Radiology Data from the Clinical Proteomic Tumor Analysis Consortium Glioblastoma Multiforme [CPTAC-GBM] collection. Cancer Imaging Arch [Internet]. 2018; Available from: https://wiki.cancerimagingarchive.net/display/Public/CPTAC-GBM#4dc5f53338634b35a3500cbed18472e0

15. Mansoor A, Bagci U, Foster B, Xu Z, Douglas D, Solomon JM, et al. CIDI-lung-seg: A single-click annotation tool for automatic delineation of lungs from CT scans. 2014 36th Annu Int Conf IEEE Eng Med Biol Soc EMBC 2014. 2014. p. 1087–90.

16. Song SH, Park H, Lee G, Lee HY, Sohn I, Kim HS, et al. Imaging Phenotyping Using Radiomics to Predict Micropapillary Pattern within Lung Adenocarcinoma. J Thorac Oncol [Internet]. Elsevier Inc; 2017;12:624–32. Available from: http://dx.doi.org/10.1016/j.jtho.2016.11.2230

17. Smith SM, Jenkinson M, Woolrich MW, Beckmann CF, Behrens TEJ, Johansen-Berg H, et al. Advances in functional and structural MR image analysis and implementation as FSL. Neuroimage. 2004. p. 208–19.

18. Jenkinson M, Beckmann CF, Behrens TEJ, Woolrich MW, Smith SM. FSL. Neuroimage. 2012;62:782–90.

19. Woolrich MW, Jbabdi S, Patenaude B, Chappell M, Makni S, Behrens T, et al. Bayesian analysis of neuroimaging data in FSL. Neuroimage [Internet]. Elsevier B.V.; 2009;45:S173–86. Available from: http://dx.doi.org/10.1016/j.neuroimage.2008.10.055
